# Esketamine and Ketamine use in the Adolescent Population for Treatment Resistent Depression: A Systematic Review

**DOI:** 10.1192/j.eurpsy.2025.1310

**Published:** 2025-08-26

**Authors:** G. Chen, Y. Li

**Affiliations:** 1 Psychiatry, Lehigh Valley Health Network, Allentown; 2 Medical School, Virginia Tech, Roanoke, United States

## Abstract

**Introduction:**

Ketamine is widely recognized for its dissociative anesthetic properties, particularly in procedural sedation and severe pain management among children, adolescents, and young adults. Despite its noted applications, research investigating ketamine’s impact on mood and suicidality within this specific demographic remains limited. This retrospective review seeks to address this gap by characterizing ketamine usage and psychiatric comorbidities in Treatment-resistant depression (TRD).

**Objectives:**

This review aims to provide an initial assessment of the safety and efficacy of esketamine in adolescents suffering from TRD with concurrent comorbidities such as GAD and SUD, and infer common drug-drug interactions that may arise from utilizing esketamine in adolescents. This preliminary overview will contribute valuable insights toward optimizing treatment strategies for this vulnerable population.

**Methods:**

This systematic review evaluates the evidence for ketamine use in children with treatment-resistant mood disorders. Two to four authors independently screened studies and extracted data on safety, tolerability, and efficacy. We searched two electronic databases for English-language studies on the therapeutic effects and side effect profiles of ketamine in youth aged 14-23 with treatment-resistant mood disorders, including those with treatment-resistant depression (with and without psychotic features) and bipolar disorder.

**Results:**

Recent studies, including a randomized, midazolam-controlled trial, indicate that IV ketamine can effectively reduce depressive symptoms in adolescents with treatment-resistant depression (TRD), with mild and manageable side effects like dissociation. No serious adverse events or instances of misuse were reported.

**Conclusions:**

In conclusion, esketamine has demonstrated effectiveness in treating treatment-resistant depression. Additionally, when combined with SSRIs, esketamine improves depressive symptoms in TRD patients, irrespective of their anxiety status. These findings highlight the potential of esketamine as a valuable treatment option for this challenging patient population. Future research will focus on optimizing dosing, exploring complementary therapies such as cognitive behavioral therapy (CBT), and identifying biomarkers for response, while considering the unique risks to the developing adolescent brain.

**Disclosure of Interest:**

None Declared

